# Biodegradable albumen dielectrics for high-mobility MoS_2_ phototransistors

**DOI:** 10.1038/s41699-023-00436-7

**Published:** 2023-11-03

**Authors:** Thomas Pucher, Pablo Bastante, Federico Parenti, Yong Xie, Elisabetta Dimaggio, Gianluca Fiori, Andres Castellanos-Gomez

**Affiliations:** 1 Materials Science Factory. Instituto de Ciencia de Materiales de Madrid (ICMM-CSIC), Madrid, 28049 Spain; 2 Departamento de Física de la Materia Condensada, Universidad Autónoma de Madrid, 28049 Madrid, Spain; 3 Dipartimento di Ingegneria dell’Informazione, Via Caruso 16, 56122 Pisa, Italy; 4 School of Advanced Materials and Nanotechnology, Xidian University, 710071 Xi’an, China; 5 Unidad Asociada UCM/CSIC, “Laboratorio de Heteroestructuras con aplicación en spintrónica”, Madrid, Spain

**Keywords:** Electronic devices, Biomaterials

## Abstract

This work demonstrates the fabrication and characterization of single-layer MoS_2_ field-effect transistors using biodegradable albumen (chicken eggwhite) as gate dielectric. By introducing albumen as an insulator for MoS_2_ transistors high carrier mobilities (up to ~90 cm^2^ V^−1^ s^−1^) are observed, which is remarkably superior to that obtained with commonly used SiO_2_ dielectric which we attribute to ionic gating due to the formation of an electric double layer in the albumen MoS_2_ interface. In addition, the investigated devices are characterized upon illumination, observing responsivities of 4.5 AW^−1^ (operated in photogating regime) and rise times as low as 52 ms (operated in photoconductivity regime). The presented study reveals the combination of albumen with van der Waals materials for prospective biodegradable and biocompatible optoelectronic device applications. Furthermore, the demonstrated universal fabrication process can be easily adopted to fabricate albumen-based devices with any other van der Waals material.

## Introduction

Two-dimensional (2D) semiconductors are promising candidates for next-generation electronics due to their ultrathin nature, exceptional electrical and optoelectronic properties, and facile material combination beyond the limits of lattice matching. Out of the huge family of semiconducting 2D materials, MoS_2_ has been exploited in several proof-of-concept devices ranging from field-effect transistors (FETs)^1–3^, to photodetectors^4–6^ and memory devices^7–10^, just to name a few. These devices have found applications in many emerging applications including neural computing, optoelectronics or the internet of things^11–13^.

Although usually underestimated, the dielectric material is also a crucial part of these devices, as their performance strongly depends on its properties. In FETs, for example, the dielectric plays a major role in their scalability, power consumption, and threshold electric field^14^. Up to now, SiO_2_ has been heavily used as dielectric in 2D-based devices, mostly because of its large availability in micro-fabrication processes but it does not provide optimal performance in 2D devices. It is therefore equally important to invest research effort in finding suitable dielectrics for 2D materials, optimized for different electronics and optoelectronics applications^15^. Pursuing high-performance devices, dielectrics like hexagonal boron-nitride (hBN), a 2D material with an atomically-flat surface and few dangling bonds, have been studied in the last years^16^. Many other insulators (2D as well as 3D) have been explored, ranging from high-k materials, like HfO_2_, ionic oxides, like CaF_2_, and recently high-k free-standing perovskites, as STO or BTO^15,17–21^.

Because of the low toxicity and cytotoxicity of MoS_2_^22,23^, it is also being proposed for biodegradable or even biocompatible electronics thus motivating the search for a biocompatible dielectric material. From the various biocompatible dielectric materials tested in the past, albumen has already shown good performance in organic FETs and memristive devices^24–26^. Albumen extracted directly from chicken eggs is mainly composed of water and different types of protein. When spin-coated and thermally treated these amino acid chains form a dielectric layer by natural denaturation^27^. The albumen integration and treatment is a rather straightforward process without the use of any filtering technique and results in dielectric thicknesses of around 500 nm. The device structure aims to extend the 2D field-effect transistor field to systems where biodegradability and biocompatibility is needed and economical waste needs to be reduced with the possibility of usage in combination with biodegradable flexible substrates^28–30^. Albumen is probably one of the simplest implementations of protein-based dielectrics with nearly endless opportunities in combination with Van der Waals materials. Compared to other bio-materials a major advantage is the resulting cost-effectiveness stemming from the fact of the simple extraction from chicken eggs without the need of chemical treatments, therefore reducing production costs. To our knowledge, nobody has yet demonstrated an integration of albumen dielectrics with van der Waals materials, which is the main goal of our work.

Here we present single-layer MoS_2_ phototransistors using biodegradable albumen as gate dielectric. We report that the fabricated devices exhibit orders of magnitude higher mobility values (*μ* ~ 5–90 cm^2^ V^−1^ s^−1^) as compared with SiO_2_-based devices (*μ* ~ 0.01–0.4 cm^2^ V^−1^ s^−1^). Moreover, we report a fabrication technique that can be applied to any other van der Waals material, and we demonstrate the optoelectronic response of the phototransistors (*R* = 4.5 AW^−1^, *t*_rise_ = 52 ms), giving a complete characterization of our albumen devices.

## Results

### Device fabrication

**Fig. 1:**
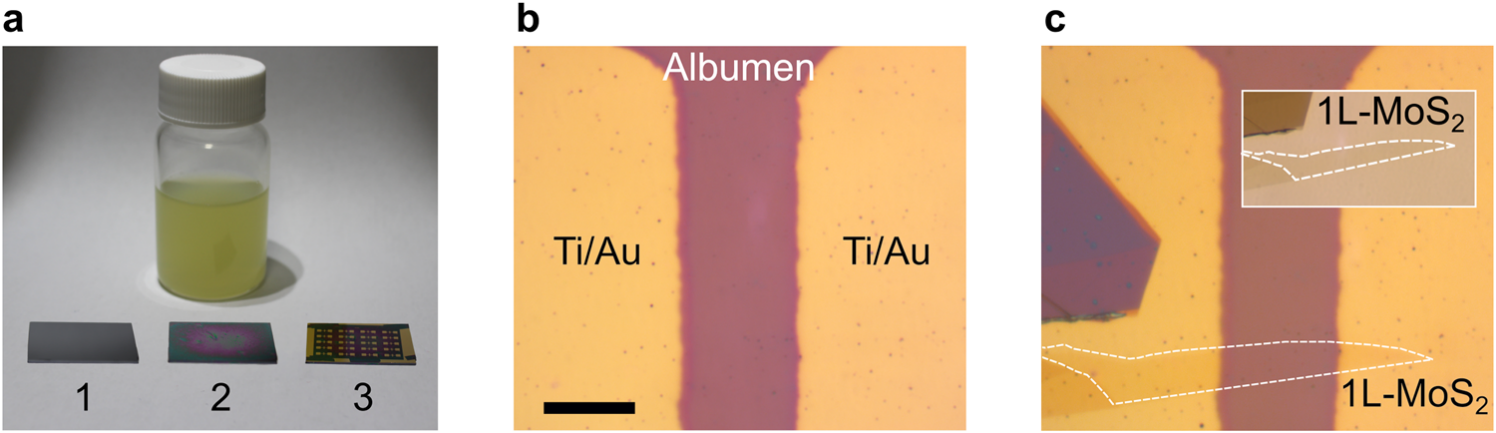
Fabrication of MoS_2_ field-effect transistors integrating eggwhite dielectric. **a** Picture of a bottle of liquid eggwhite and the three main processing stages of the substrate in chronological order: a bare 2 × 1 cm^2^ silicon substrate (1), a substrate with spin-coated and baked eggwhite thin film (2), and a substrate with eggwhite dielectric after electrode evaporation (3). **b** Optical micrograph of drain and source electrodes evaporated with a shadow mask onto the eggwhite dielectric. The scale bar is 20 µm. **c** Optical micrograph of the electrodes shown in (**b**) after transferring a single-layer MoS_2_ bridging the electrodes to create the semiconducting channel. Inset: Transmission mode optical microscopy image of the MoS_2_ flake on the Gel-Film stamp ready for characterization and subsequent dry-transfer.

The albumen-based transistors are fabricated by drop-casting and spin-coating liquid albumen onto a heavily doped silicon substrate followed by baking on the hot plate in two steps of 10 min at 100 °C and 120 °C. Different albumen layer thicknesses can be obtained by changing the spin-coating speed (Supplementary Fig. 1, Supporting Information). For better device stability two layers of albumen were spin-coated on top of each other, following the same baking steps after each coating and resulting in an overall thickness of ~500 nm (Fig. 1a, sample 2). Subsequently, Ti (5 nm)/Au (45 nm) source-drain electrodes were deposited by e-beam evaporation through a shadow mask (Ossila, E321). The result is shown in Fig. 1a, sample 3. Figure 1b shows an optical image of the gap between the source-drain contacts deposited onto the albumen layer. The heavily doped silicon substrate underneath is used as a backgate electrode.

A single-layer MoS_2_ flake was mechanically exfoliated from a bulk molybdenite crystal (Molly Hill Mine, Quebec, Canada) onto a Gel-Film stamp (Gel-Film WF 4 × 6.0 mil by Gel-Pack), followed by a dry deterministic transfer to build the channel between the Ti/Au electrodes^31,32^. Before transferring, the flake was characterized and identified as a monolayer by differential reflectance measurement using a homebuilt micro-reflectance setup^33^. The differential reflectance measurement including a comparison to another multilayer flake is given in the supporting information (Supplementary Fig. 2). Interestingly, we found that the MoS_2_ adhesion to albumen and gold was found to be even larger than that observed for devices featuring SiO_2_ dielectric, and the devices showed a very high conductivity even right after transfer (without the need of an additional annealing step). This is in striking contrast to SiO_2_-based MoS_2_ transistors where we have observed that after deterministic transfer the device conductivity was rather poor and a vacuum annealing step is required. Raman and photoluminescence measurements are proven tools to check the properties of 2D materials^34–36^. These measurements confirm that MoS_2_’s material properties are preserved after transfer on top of albumen. No changes in peak positions are observed compared to data taken from a SiO_2_ substrate, excluding a possible functionalization of the 2D material (Supplementary Fig. 3)^37,38^.

To better understand the function of the albumen insulating layer it is beneficial to review its behavior during processing. The albumen is made up of about 90% water and 10% proteins. The application of heat starts two commonly known processes: denaturation and coagulation. Heating alters the amino acid chains by partially or completely unwinding the protein. This process is called denaturation. Once a protein has denatured, it may meet and bind with another denatured protein. When a sufficient number of denatured proteins are added together, we are dealing with a coagulation phenomenon. In the egg, the coagulated proteins form a three-dimensional layer that traps and immobilizes water. The most important chemical reaction is the formation of disulfide bonds, between two cross-linked protein molecules^27^. The protein disulfide bonds reduce the gate leakage current in the dielectric layer of albumen FETs^39^. Interestingly, this cross-linked protein structure makes the albumen dielectric very sturdy. We have found that, after baking, the albumen layer can withstand acetone and 2-propanol treatment and remains electrically stable with no significant change in leakage current. This opens up the possibility of employing a lithography/lift-off process on top of the albumen, thereby enabling a wide range of post-processing techniques. We address the reader to Supplementary Figs. 4 and 5 for details about the marker lithography process.

### Electrical analysis

**Fig. 2:**
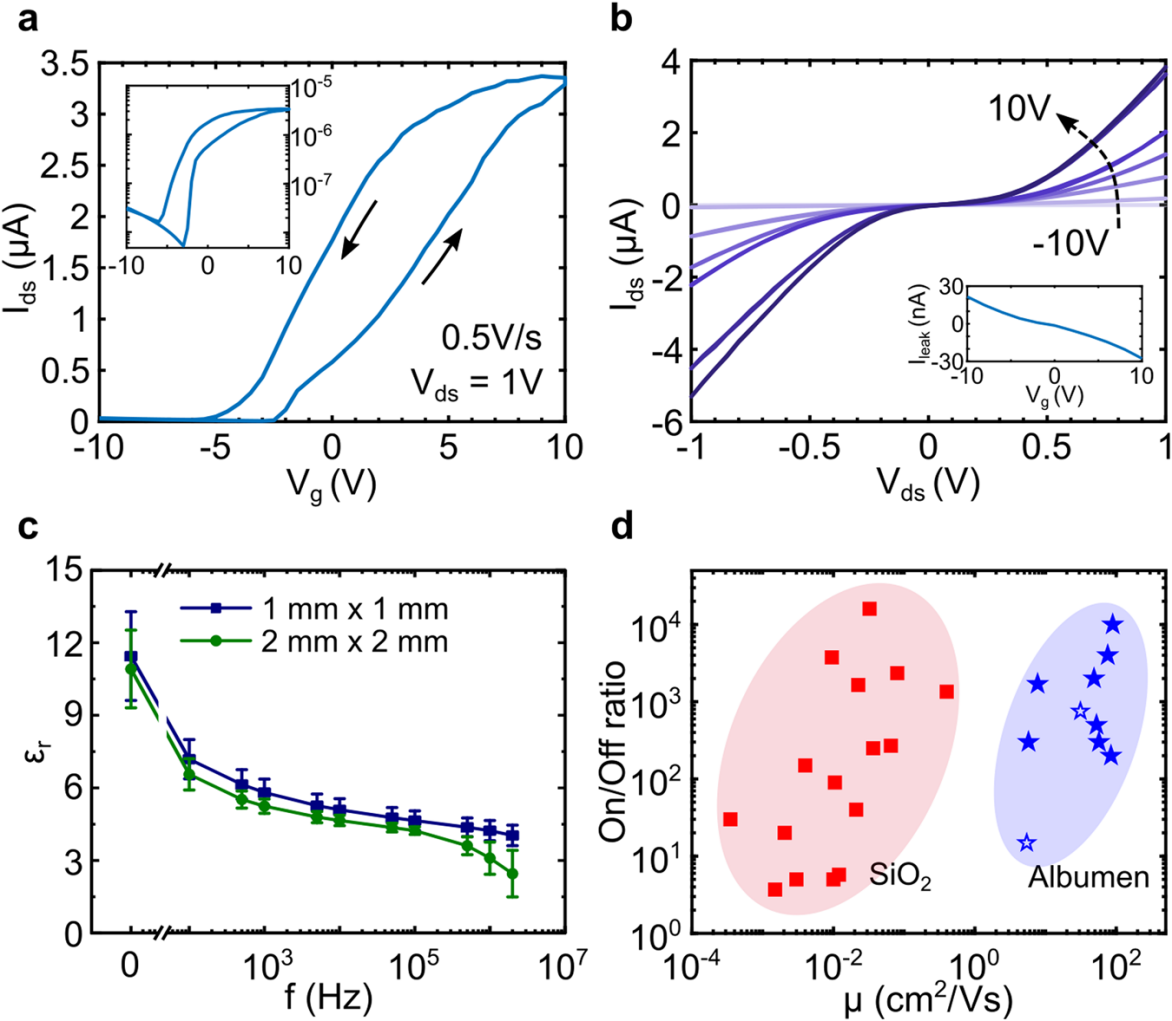
Electrical device characterization of MoS_2_ field-effect transistors with eggwhite dielectric. **a** Transfer curves (*I*_sd_ vs. *V*_g_) plotted in linear and semi-logarithmic (inset) fashion. **b** Corresponding current vs. voltage curves (*IV*s) for different gate voltages ranging from −10 to 10 V. The inset shows gate leakage current for the used gate voltage range. **c** Relative dielectric constant plotted as a function of frequency for 1 mm^2^ area (blue) and 4 mm^2^ area (green) albumen capacitors. Error bars represent standard deviation of the dielectric constant values. The graphs report mean values (arithmetic) and standard deviation couples. **d** An overview of all fabricated albumen-based transistors (blue) in comparison to SiO_2_-based transistors (red). The albumen-based devices show significant higher mobility values and similar ON/OFF current ratios than SiO_2_-based ones. Full stars correspond to devices fabricated with bottled pasteurized eggwhite and empty stars to the ones made out of eggwhite extracted from whole chicken eggs.

Electrical and optical device characterization were carried out using a homebuilt probe-station setup under ambient conditions, allowing to perform current *vs*. voltage curve characterization (*IV*s hereafter), transfer curve measurements and photocurrent measurements. From these, we can extract all necessary figures of merit for comparison with other 2D transistors and phototransistors. In general, the fabrication and measurement of devices showed an exceptional reproducibility with high yield in working devices. Figure 2a, b depicts the electrical characterization for one albumen transistor as an example of electrical characterization. Gate voltage sweeps are comparably small for all devices in the range of −10 to 10 V, giving high tunability even with moderate low gate voltage values. The transfer curve (displayed in a linear and logarithmic way in Fig. 2a) illustrates a high on-current. A clear counter-clockwise hysteresis is observed in all measured albumen devices, ascribed to the dielectric itself. As shown in other reported works, this counter-clockwise hysteresis is attributed to an occurring polarization within the albumen layer^25,40^ and can be potentially lowered by controlled moisture and oxygen levels of the measurement setup^26^. The inset in Fig. 2b depicts the leakage current through the albumen layer, proofing the negligible contribution to the device performance. In addition, a more detailed leakage study confirms that the albumen is electrically stable in a wide range of thicknesses and common humidities (Supplementary Fig. 5). From the recorded data one can extract the low-field field-effect mobility by using the transfer curve slope and the expression

1 $$\mu =\left[\frac{\partial {I}_{{\rm{sd}}}}{\partial {V}_{{\rm{g}}}}\right]\times \left[\frac{L}{W\cdot C{^\prime} \cdot {V}_{{\rm{sd}}}}\right],$$

where *L* is the channel length, *W* is the channel width and *C* is the dielectric capacitance per unit area. We performed capacitance measurements on multiple albumen films with different contact areas as a function of frequency (Fig. 2c). By using the parallel plate capacitor structure, we obtain a dielectric constant of *ε*_r_ = 11.2 ± 1.9. This value is slightly higher as compared to other reported values^26^, from which we calculate a capacitance per unit area *C’* = 13.7 nF cm^−2^ (for more details regarding the full capacitance measurement from DC up to 1 MHz see Supplementary Figs. 6 and 7). This allows us to extract mobility of *μ* = 52.5 cm^2^ V^−1^ s^−1^ for the device shown in Fig. 2a. We also confirmed that the dielectric constant values of the albumen layer do not change with thickness in the range of thicknesses used in all devices, justifying the use of one dielectric constant for all calculations.

The detailed capacitance analysis shows that the average capacitance values tend to decrease with increasing frequency and is maximum at DC. We also found that the parallel conductance of the albumen dielectric is up to one order of magnitude higher than that expected for an ideal solid-state capacitor. These two observations are compatible with the presence of ionic current within the albumen, and thus the formation of an electric double layer, typical of capacitors based on ionic materials^41,42^. This mechanism would explain the observed high mobility in the albumen-based MoS_2_ transistor as it has been reported that ionic gating can improve the carrier mobility in MoS_2_ reaching up to ~100 cm^2^ V^−1^ s^−1^ at room temperature, inducing ionic gating rather than solid-state electric field effect^43–45^. Schottky contact behavior can be observed in Fig. 2b, resulting from the Au/MoS_2_ interface. However, according to other works ionic gating allows to largely dope the MoS_2_ channel, thus reducing the Schottky barrier and allowing high-mobility values^43^. To confirm a reduced Schottky barrier we have performed and compared in-operando Kelvin probe force microscopy measurements (KPFM) of an MoS_2_-flake on top of albumen and SiO_2_ under different source-drain bias voltages. Visualizing the potential drop from electrode to electrode over the MoS_2_ channel and subtracting the recorded zero-bias potential to remove the work function difference from the electrostatic profile, allows us to extract the Schottky barrier for both cases. We observed half of the barrier height on top of albumen compared to SiO_2_. The results still show a noticeable Schottky barrier, hence explaining the nonlinear *IV* curves. We forward the reader to the Supporting Information for the details of those measurements.

The mobility values for all fabricated devices can be calculated and plotted together with the corresponding ON/OFF current ratios (Fig. 2d). Device parameters and extracted values, including transconductance and carrier concentration values, as well as all *IV* curves and transfer curves are given in the supporting information (Supplementary Table 1 and Supplementary Figs. 9–16). Apart from consistent counter-clockwise hysteresis, the measured transfer curves show device-to-device variations stemming from the fact that devices have been fabricated with different batches of albumen sources of same origin without filtering techniques. The calculated mobility values for all albumen devices are in the range of 5–89 cm^2^ V^−1^ s^−1^ which is comparably high with respect to standard single-layer MoS_2_ transistors with SiO_2_ dielectric, as expected for ionic-gated devices. To further proof the significant increase in mobility we fabricated multiple transistors choosing SiO_2_ as the insulating material, following the same protocol as for the albumen-based transistor. SiO_2_ is still the most commonly deployed dielectric material in 2D semiconductor devices due to its widely usage in semiconductor industry. To ensure a comprehensive outcome the same materials and processing steps were employed for the SiO_2_-based transistors, simply changing the substrate to Si/SiO_2_ with an oxide thickness of 285 nm. After performing the same electrical measurements with the SiO_2_ devices, clearly knowing its dielectric constant (*ε*_r_ = 3.9), the values for ON/OFF current ratios and mobilities can be calculated. These values are plotted next to the albumen device values (Fig. 2d). SiO_2_-based transistor device parameters and selected transfer curves in similar fashion to the albumen devices are given in the supporting information (Supplementary Table 2 and Supplementary Fig. 17). It is clearly shown that the albumen transistors exhibit orders of magnitude higher mobility values by keeping the same range of ON/OFF current ratios compared to the SiO_2_ counterparts. Surprisingly this is true for all fabricated devices with absolutely every albumen transistor outperforming any SiO_2_ one, although all the SiO_2_ devices have been thermally annealed in vacuum conditions at 200 °C for at least 2 h prior to measuring, which was not needed for the albumen devices. It is important to note, that all devices were measured at ambient conditions in a two-terminal setup configuration, without removing contact resistance. Hence the calculated mobility reported here are lower bound estimates which explains why our SiO_2_-based device mobilities are comparably low to what is reported in other works^1,46,47^. It is worth noting, that all devices are nonencapsulated and therefore very sensitive to ambient conditions (as well seen with similar values in other works^46,48,49^), explaining the poor mobility values and further highlighting the improved characteristics of the albumen devices. We have observed that the mobility of SiO_2_-based MoS_2_ transistors quickly degrade upon air exposure after vacuum annealing. As it is beyond the scope of the current publication, it will be reported elsewhere. All SiO_2_-based devices show typical clockwise hysteresis, which once again supports the claim of attributing the counter-clockwise hysteresis of the albumen devices to the dielectric itself.

### Optoelectronic analysis

**Fig. 3:**
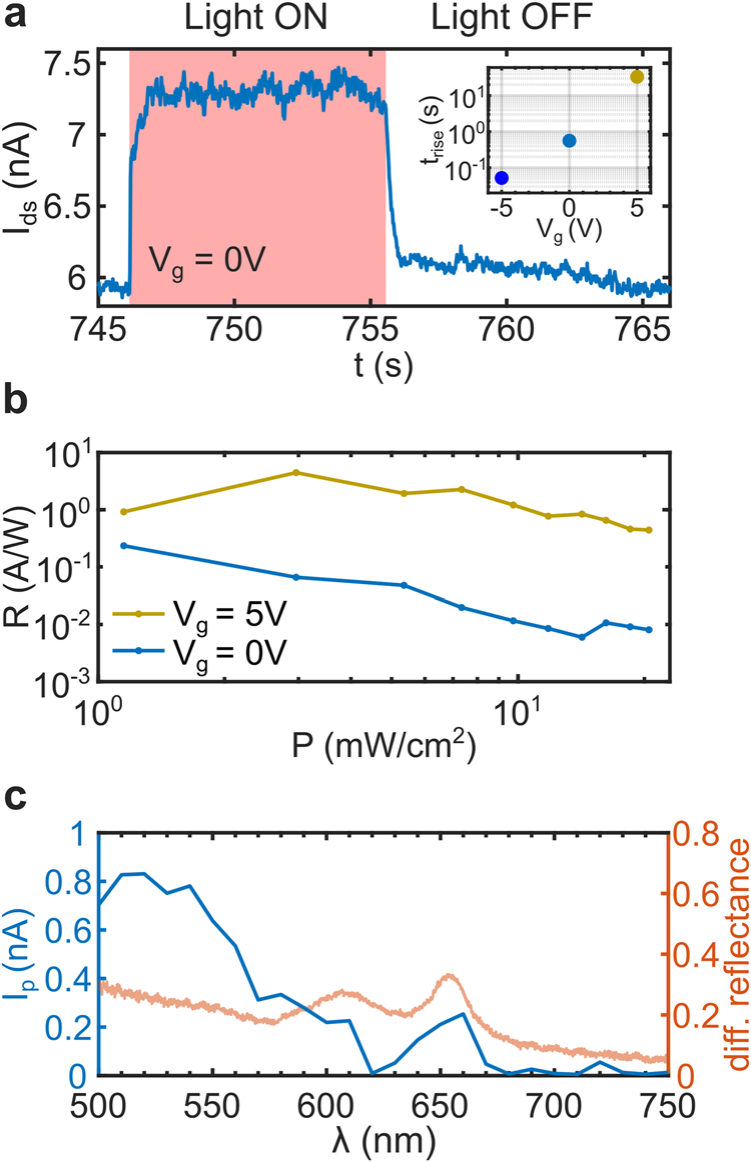
Optoelectronic characterization of MoS_2_ field-effect transistors integrating eggwhite dielectric. **a** Photoresponse under pulsed LED illumination of 660 nm wavelength (*V*_g_ = −5 V, *V*_bias_ = 1 V, LED power density 75.6 mW cm^−2^). On and off illumination times are 10 s. Inset: Rise time, extracted from measurements under pulsed illumination like that shown in (**a**), as a function of the gate voltage. **b** Responsivity as a function of the illumination power density under 660 nm LED emission (*V*_bias_ = 1 V). **c** Wavelength dependence of the photocurrent showing an enhanced photoresponse at MoS_2_’s excitonic energies (*V*_bias_ = 1 V). For comparison the measured differential reflectance spectrum of the MoS_2_ flake prior transfer is added to the plot, relating A (660 nm) and B (610 nm) exciton peaks to the peak responses in photocurrent measurements.

As MoS_2_ is also a promising material for optoelectronic applications, it is of interest to test the response of the albumen-based devices to external illumination. Figure 3a shows the photocurrent of an albumen-based MoS_2_ device, at zero gate voltage, for an illumination with a 660 nm LED light switched On (10 s) and Off (10 s), displaying rather steep rise/fall times. Changing the gate voltage leads to a remarkable change in the rise/fall times, as increased doping level is known to lead to a dominant role of photogating photocurrent generation mechanism. This effect can be described by the two main mechanisms contributing to the photoresponse of an MoS_2_-based device: the photogating effect (higher responsivity, slower response) and the photoconductive effect (lower responsivity, faster response), where the first one has been attributed to a shift in threshold voltage of the transistor and the second one to hole trapping^50–52^. For negative gate voltages, where the dominant photocurrent generation mechanism is the photoconductive effect, the responsivity is lower, but the rise/fall times becomes much faster. The inset in Fig. 3a displays this behavior with a measured rise time of 54 ms under a gate bias of −5 V and an increase in response time to 33 s for a positive gate voltage of +5 V. Although a positive gate voltage means a slower device response it also gives rise to better responsivity values due to the dominant photogating effect. The power-dependent responsivity values of the device for gate voltages of zero and +5 V are shown in Fig. 3b. The transistor’s responsivity to light rises as the gate bias is increased, with a peak of 4.5 AW^−1^ at *V*_g_ = +5 V and a LED power density of 2 mWcm^−2^. This responsivity value can be considered high compared to other studies of MoS_2_-flake-based phototransistors, usually ranging between 0.008 and 0.57 AW^−1^, whereas response times are in the same range^48,53^. Reports with higher responsivity values make use of more sophisticated device structures, much higher bias- and gate voltages, or multilayer MoS_2_ configurations^4,54–56^. We also characterized the spectral response of our albumen-based devices by measuring the photocurrent while sweeping the wavelength over the 500–750 nm range. This measurement shows two prominent peak responses at A- and B-exciton energies of MoS_2_ (Fig. 3c) followed for a vanishing photocurrent for illumination with energies lower than the A exciton. For better illustration the same graph shows the differential reflectance measurement of the monolayer flake prior to transfer on the Gel-Film stamp, distinctly displaying its excitonic response and additionally confirming the single-layer thickness.

At this point it would be natural to wonder what would happen if one used the whole egg as a dielectric instead of just the albumen. For that reason, we fabricated another set of devices applying the same process steps, but this time using a mixture of albumen and yolk as the dielectric layer. The resulting devices still show transistor behavior both in electrical and optoelectronic characterization, but expectedly exhibit a lot of gate leakage and therefore perform not like the albumen-based ones. We forward the reader to the Supplementary Fig. 18 for details about the whole egg MoS_2_ transistors.

## Discussion

In summary, we present the use of albumen dielectrics in combination with single-layer MoS_2_ to realize field-effect transistors, as a first step to biocompatible device applications with semiconducting van der Waals materials. Compared to traditional SiO_2_-dielectric based devices, we not only confirmed a higher dielectric constant (*ε*_r_ = 11.2 ± 1.9), but the results also indicate a major advantage in mobility values, with mobilities reaching up to ~90 cm^2^ V^−1^ s^−1^. Optoelectronic device characterization revealed responsivities of *R* = 4.5 AW^−1^ and rise times of *t*_rise_ = 52 ms. Furthermore, the fabrication procedure demonstrated can be applied in any other combination with van der Waals materials due to its universal nature. Albumen’s resistivity to chemicals used in standard lithography and lift-off processes additionally opens a world of post-processing techniques that can even lead to more complex devices. Our work demonstrates the combination of albumen dielectrics with two-dimensional van der Waals materials, introducing a new platform for biodegradable 2D applications.

## Methods

### Materials and albumen dielectric fabrication

Liquid eggwhite was prepared unfiltered for spin-coating from two different sources: from bottled pasteurized eggwhite (Mercadona, Huevos Guillén S.L.) and from whole chicken eggs after separation of yolk and eggwhite using a kitchen strainer. MoS_2_ flakes were obtained from natural molybdenite mineral (Molly Hill Mine, Quebec, Canada) by mechanical exfoliation using Nitto tape (Nitto SPV 224).

Eggwhite typically consists of liquid and coagulated albumen. Only the liquid part is used for the spin-coating process, as it results in a thinner and more homogenous film. The unfiltered liquid eggwhite was drop-casted and spin-coated onto the silicon substrate using a rate of 4000 rpm for 40 s. Subsequently, it was baked on a hot plate at 100 °C for 10 min and 120 °C for 10 min. If necessary, the procedure can be repeated for a second layer and higher rpm, resulting in a more stable insulating layer.

### Electrode deposition

Prepatterned gold electrodes were fabricated by evaporation of 5 nm Ti (used as an adhesion layer) and 45 nm Au onto a SiO_2_ (285 nm)/Si (p + +) substrate through a shadow mask (Ossila, E321) in an electron-beam evaporator system.

### Electrical characterization

All electrical characterizations of devices were carried out in a homebuilt measurement setup under ambient conditions. A source-meter unit (Keithley 2450) was used for performing the electrical measurements between source and drain electrodes. Two TENMA programmable benchtop power supplies (model 72–2715) are connected in parallel to perform gate voltage sweeps between −10 and +10 V.

### Optoelectronic characterization

The photoresponse of the albumen devices was tested with a homebuilt probe station operating at room temperature in ambient conditions. The system is connected to a Keithley 2450 source-measure unit to measure the electrical transport. For the optical excitation, we recur to multimode fiber-coupled light sources, attached to the optical inspection system. This allows us to produce a circular spot of 850 μm in diameter with homogeneous power density over the sample. We employed one LED source (Thorlabs, MxxxFy series) of 660 nm wavelength to study the power-dependent photocurrent generation and a Xenon lamp with an integrated monochromator (Bentham TLS120Xe) to test the photoresponse at different illumination conditions.

### KPFM characterization

A commercial atomic force microscopy (AFM) system, from CSI Instrument, operating in ambient conditions was employed to perform morphological and surface potential (KPFM) characterization of the samples. Measurements have been acquired in dynamic mode, using the amplitude as the feedback channel for topography acquisition. Surface potential maps have been measured using single pass KFM mode (HD-KFM) with PtSi-coated tip from Nanosensors (PtSi-FM). Vac voltage for KPFM measurements was applied to the tip, *V*_ac_ = 1 V. Image analysis was performed with the Gwyddion free software. A Keithley 2450 source-measure unit was used to apply different bias voltages between the Au electrodes.

### Supplementary information

Supplementary Information

## Data Availability

The data of this study are available from the corresponding author upon reasonable request.
