# Supplementary material for: Biodegradable albumen dielectrics for high-mobility MoS2 phototransistors
Source: NPJ 2D Mater Appl. 2023 Nov 3;7(1):73. doi: 10.1038/s41699-023-00436-7 (PMC11041700; doi:10.1038/s41699-023-00436-7)
Supplement: Supplementary file 1 — Supplementary Information [file 41699_2023_436_MOESM1_ESM.pdf]

Supporting Information:

Biodegradable albumen dielectrics for high-mobility MoS_2 phototransistors

Thomas Pucher[1], Pablo Bastante[2], Federico Parenti[3], Yong Xie[1,4], Elisabetta Dimaggio[3], Gianluca Fiori[3] and Andres Castellanos-Gomez[1,5*]*

[1] *Materials Science Factory. Instituto de Ciencia de Materiales de Madrid (ICMM-CSIC), Madrid, 28049, Spain.*

[2] *Departamento de Física de la Materia Condensada, Universidad Autónoma de Madrid, 28049, Madrid, Spain*

[3] *Dipartimento di Ingegneria dell'Informazione, Via Caruso 16, 56122, Pisa, Italy*

[4] *School of Advanced Materials and Nanotechnology, Xidian University, Xi'an 710071, China*

[5] *Unidad Asociada UCM/CSIC, "Laboratorio de Heteroestructuras con aplicación en spintrónica"*

thomas.pucher@csic.es

andres.castellanos@csic.es

To do a fast determination of albumen thickness we use differential reflectance measurements with a spectrometer and Fresnel's equations to fit the measured data. The refractive index of eggwhite is 1.5, which allows to calculate the optical contrast using a reflectance measurement of the bare silicon substrate and a reflectance measurement of the spin-coated and baked eggwhite. By fitting the optical contrast to the calculated Fresnel contrast we can easily and quickly determine the albumen layer thickness. We used different spinning speeds and determined each thickness with the spectrometer measurements. Afterwards we did AFM thickness measurements to compare these values to the fitted ones and get a very good agreement with the calculated data, which justifies the application of the fast contrast method. Figure S1 gives thickness values for the range 3000-6000 rpm measured with AFM and fitted with the spectra, as well as exemplary contrast fits for 3k and 6k spinning speeds.

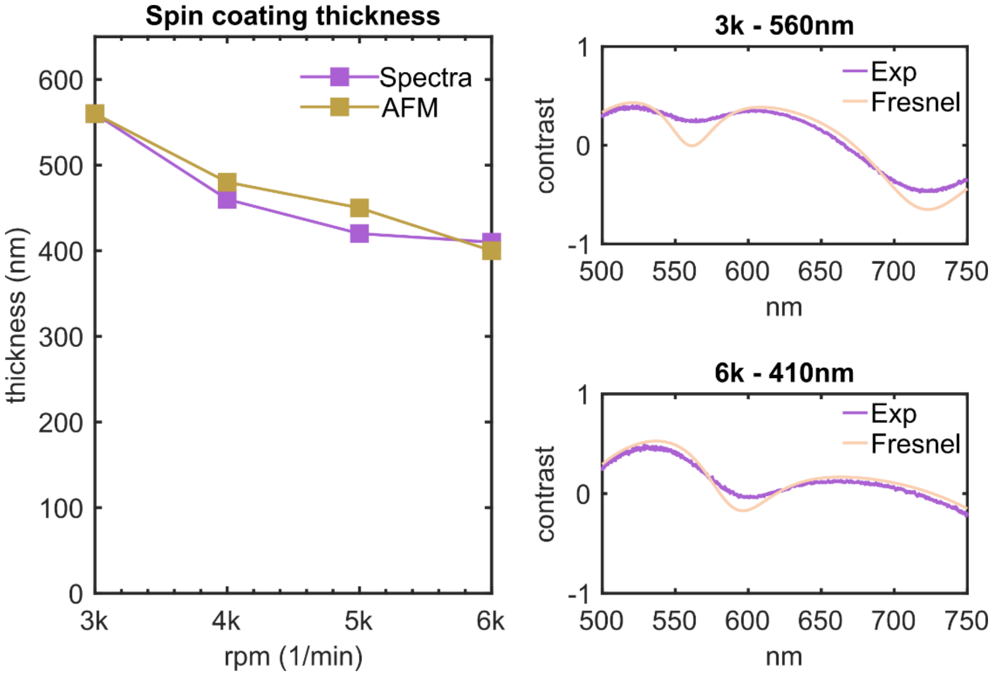

Supplementary Figure 1: Albumen film thickness determination methods. Different spin-coating speeds are compared by reflectance spectrometer measurements and AFM measurements, proving the reliability of fast spectrometer thickness determination. The reflectance data is fitted using Fresnel's equations. The fits for two spin-coating speeds (3000 and 6000 rpm) are shown on the right, giving a good agreement between experimentally measured reflectance and the expected Fresnel curves.

To confirm the monolayer thickness of the used flakes we measure the different thicknesses of a multilayer flake for comparison in Supplementary Figure 2. The used monolayer of albumen transistor #18 is depicted in Supplementary Figure 2a and its corresponding differential reflectance measurement in Supplementary Figure 2b. A multilayer flake for comparing measurements is shown in Supplementary Figure 2c. Three different layers of this flake were measured, monolayer (1L), bilayer (2L) and three-layer configuration (3L). Comparing the reflectance measurement of our used flake and the measurements obtained from the multilayer flake, it is clear that we can easily identify our flakes as monolayers prior to transfer. Note that the bilayer measurement illustrates the existence of the interlayer exciton, confirming further our point.

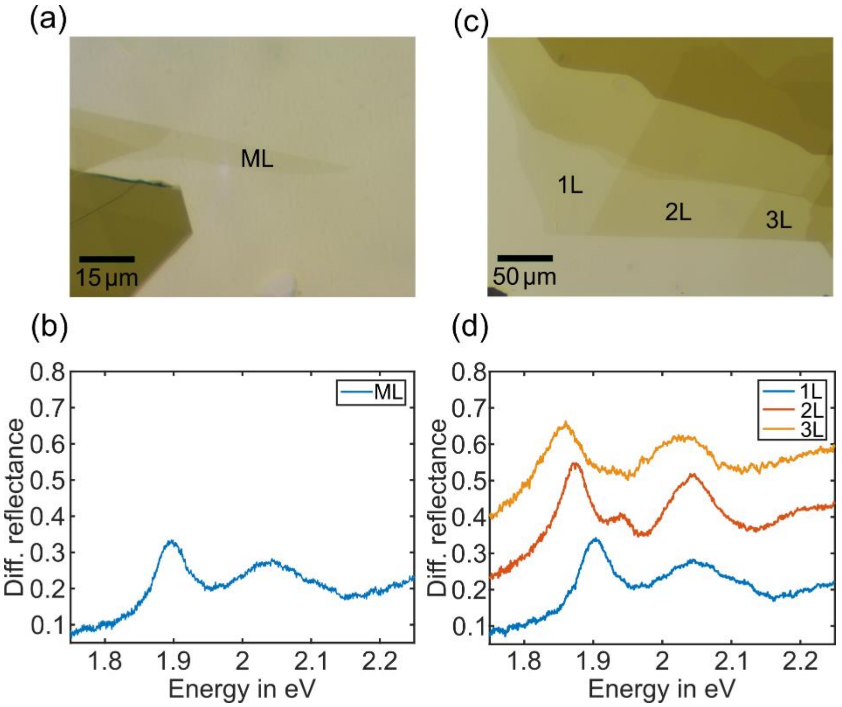

Supplementary Figure 2: Differential reflectance measurements of MoS_2 layers. (a) Optical microscope image of monolayer MoS_2 before transfer on the albumen substrate. (b) Differential reflectance measurement of flake in (a). (c) Optical microscope image of MoS_2 flake with multiple layer thicknesses. (d) Differential reflectance measurements of monolayer (1L), bilayer (2L) and three-layer part (3L) of flake from (c).

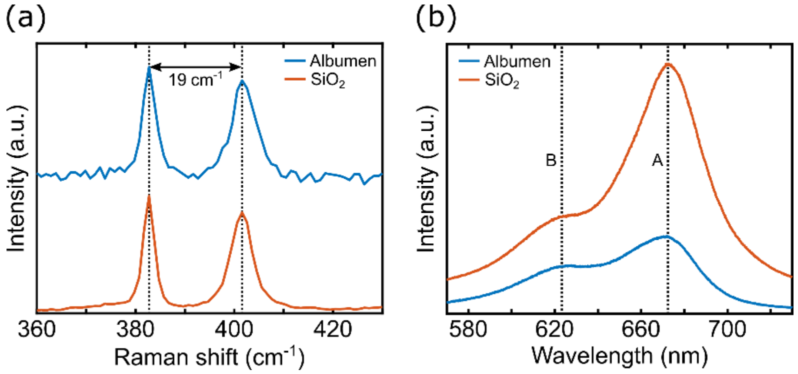

Supplementary Figure 3: Raman and PL measurements of MoS_2 layers. (a) Raman spectroscopy characterization of monolayer MoS_2 on an albumen substrate and a SiO_2 substrate. (b) Photoluminescence characterization of the same monolayers MoS_2 for each substrate. The excitation wavelength of the system is 532 nm.

For demonstration purposes an easy marker-process was tested, using a commercial marker pen to apply an evaporation mask on a processed substrate with an albumen layer on top. The mask was created by simply drawing a line with the marker pen. In the following step a layer of 50 nm gold was evaporated onto the substrate. For the lift-off the substrate was left in acetone for 10 min, rinsed with 2-propanol and blow-dried with a nitrogen gun. Electrical leakage characterization was carried out to confirm the stability of albumen after immersion in Acetone. A sample of albumen with top gold contacts was immersed in Acetone and rinsed in Isopropanol for different times with leakage current measurements after each immersion. The albumen layer does not get damaged by the lift-off process (Supplementary Figure 5c).

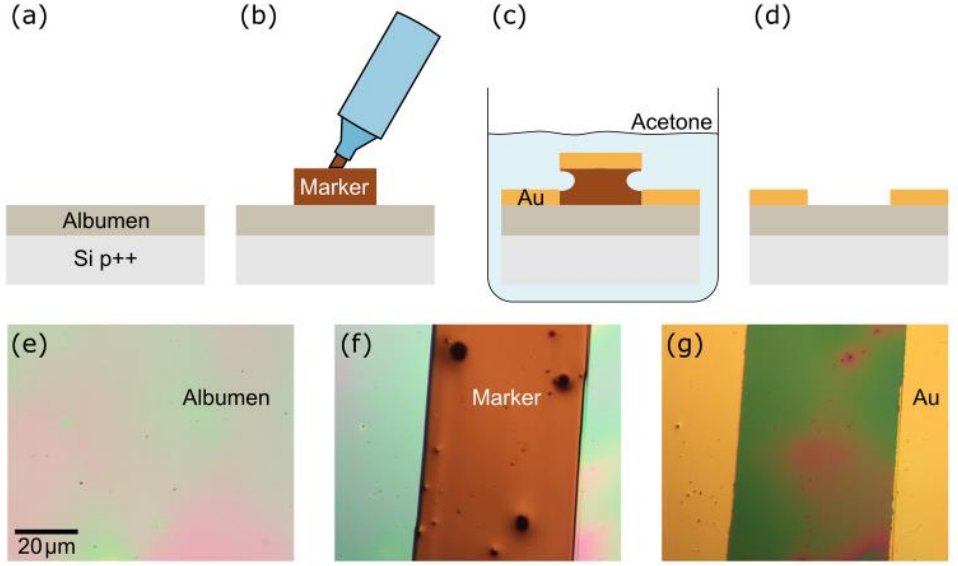

Supplementary Figure 4: Marker lithography process. Panels (a)-(d) show the used procedure to realize patterned gold films on albumen films, making use of albumen's resilience against acetone and 2-propanol. (a) Spin-coating and baking of a 300 nm thick albumen film on a Si substrate. (b) Structuring desired patterns using a commercial marker pen with a tip width of 0.75 mm. After the pattern is applied a 50 nm layer of gold is evaporated using an electron beam evaporation system. (c) Lift-off in an acetone bath. (d) The gold layer is structured while the albumen layer is unharmed. Microscope images of different steps show the albumen layer before processing (e), the albumen layer covered with a marker line (f) and the substrate after lift-off, demonstrating the intact albumen film and patterned gold structures.

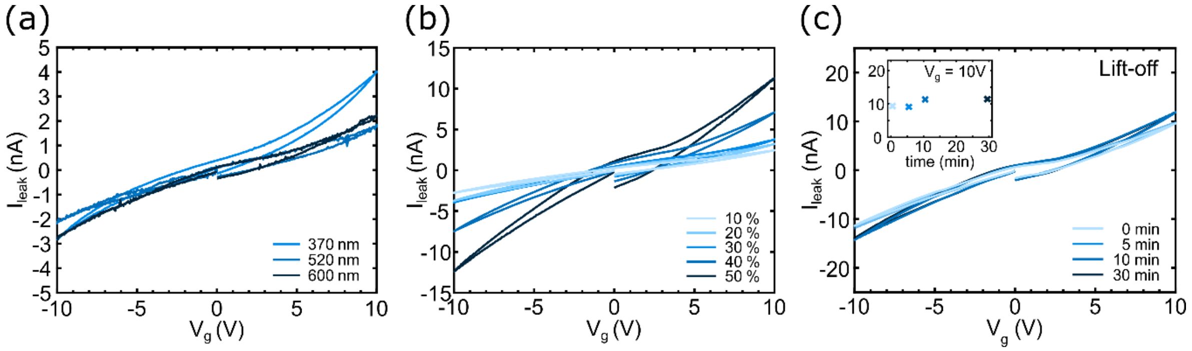

Supplementary Figure 5: Leakage current characterization. (a) Thickness dependent leakage current of one series of albumen samples from the same egg with thicknesses ranging from 370 nm to 600 nm. (b) Humidity dependent leakage current for an albumen layer of 550 nm thickness in the range of 10-50 % humidity. (c) Changes of leakage current for an albumen layer of 550 nm thickness for different immersion times in Acetone and subsequent rinsing in Isopropanol are shown up to a time of 30 min. The inset shows the maximum leakage current values for under a bias of 10 V. All samples have an effective electrode area of 1 x 1 mm^2.

To fully understand the electrical properties of the albumen dielectric, several capacitors with different active areas have been defined. The albumen capacitors were fabricated on a highly doped silicon substrate, which acts as the bottom electrode, and exploiting a top electrode with evaporated gold contact. The electrical characterization has been performed modelling the capacitor with an R_p-C_p parallel model as shown in Supplementary Figure 6a, with R_p taking into account the leakage through the dielectric. The effects of the series resistance were neglected, considering the high conductivity of the electrodes.

C_p and R_p values have been estimated by characterizing the capacitors as a function of frequency, down to the DC. In particular, the AC measurements have been performed with an LCR meter (KEYSIGHT E4989A) using a four-probe configuration, in the frequency range 100Hz - 2MHz, while applying a sinusoidal signal with amplitude equal to 1 V. An

open circuit calibration has been performed before each data acquisition to ensure measurement accuracy.

For the DC characterization, the circuit shown in Supplementary Figure 6a has been exploited, where the Device Under Test (DUT) has been connected between the inverting input and the output of a low-noise operational amplifier (Op-Amp) (LF356N). The chosen amplifier also ensures that the bias current generators can be neglected with respect to the bias current I_p of the DUT, which is further generated by the series of a DC voltage V_p and the resistor R = 10 MΩ (i.e., $I_p = V_p/R$). Before the measurement, the switch is closed, so that the current I_p flows through the switch. At t = 0 s, the switch is opened, and I_p flows through the DUT. The generated voltage transient across the DUT, and hence at the output, has been monitored by means of an oscilloscope connected to the output of the circuit.

The values of the capacitance and of the resistance have been estimated, fitting the output characteristic through the function:

$$f(t) = I_p R_p \left(1 - e^{-t/R_p C_p}\right), \quad (1)$$

as indicated in Supplementary Figure 6b. For the characterization, DC voltage values between 0.4 V to 0.8 V have been chosen. An open circuit calibration has been performed before each data acquisition in order to evaluate the parasitic capacitance of the system, which can be considered as an offset in the capacitor measurements.

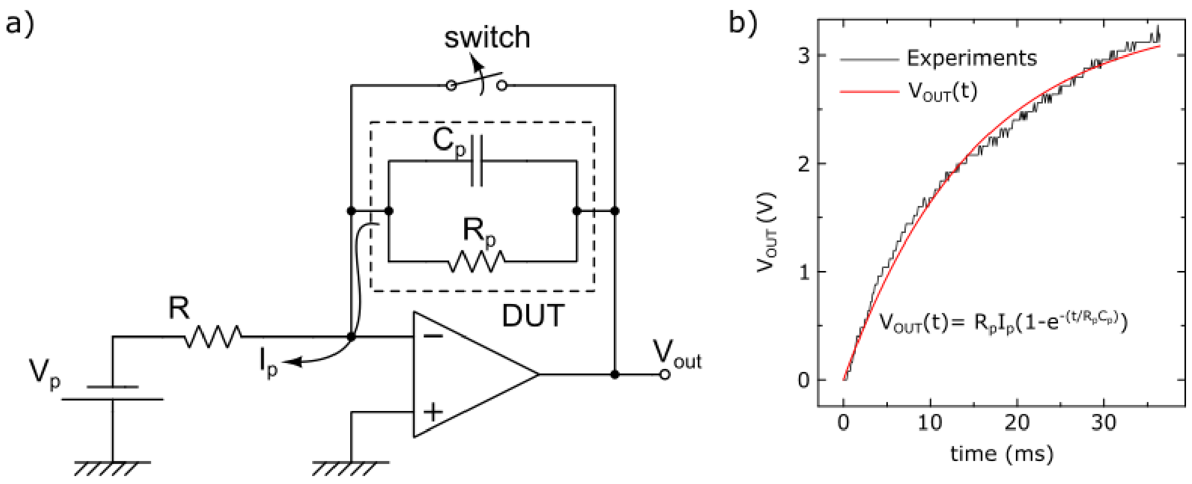

Supplementary Figure 6: (a) Electric circuit used for the DC characterization of the capacitor, considered as the parallel of a resistor R_p and a Capacitance C_p. (b) R_p and C_p are extracted through a fitting procedure of the output voltage V_{out} as a function of time with the analytical formula shown in figure.

The results of the AC and DC characterizations as a function of frequency are shown in Supplementary Figure 7. To evaluate the dispersion of the device characteristics, several capacitors have been measured, with areas of 1 mm x 1 mm and 2 mm x 2 mm. Supplementary Figure 7a & 7b show the capacitance and the parallel conductance values (G_p = $1/R_p$) per unit area, as a function of the frequency. Supplementary Figure 7c shows the values of the ratio between the capacitive susceptance and the parallel conductance: this ratio is an indication of the quality of the capacitors, i.e., a higher ratio represents a device closer to the ideal, where the leakage through the dielectric can be neglected. Generally, this value is not very high, indicating that losses in the devices are not negligible. This is expected in a capacitor based on an ionic conductor material where ion current would explain the high parallel conductance.

Supplementary Figure 7a shows that the average capacitance value tends to decrease with increasing frequency and is maximum at DC: this trend might be compatible with the presence of an electric double layer, typical of capacitors based on ionic materials.

The dielectric constant values as a function of the frequency were derived using the parallel plate capacitor equation:

$$\varepsilon_r = {C_p t}/{A\varepsilon_0}, \quad (2)$$

where A is area of the capacitor, ε_0 is the vacuum permittivity (8.85×10^{-12} $AsV^{-1}m^{-1}$), while an average thickness (t) equal to 725 nm of the albumen dielectric layer has been considered. Additionally, another batch of albumen capacitors with different thicknesses was measured to evaluate the effect of dielectric thickness on the capacitance and dielectric constant. The plots show that in a thickness range of 370-600 nm the dielectric constant does not depend on the thickness of the albumen layer. Especially the DC dielectric constant values are showing no change depending on the thickness.

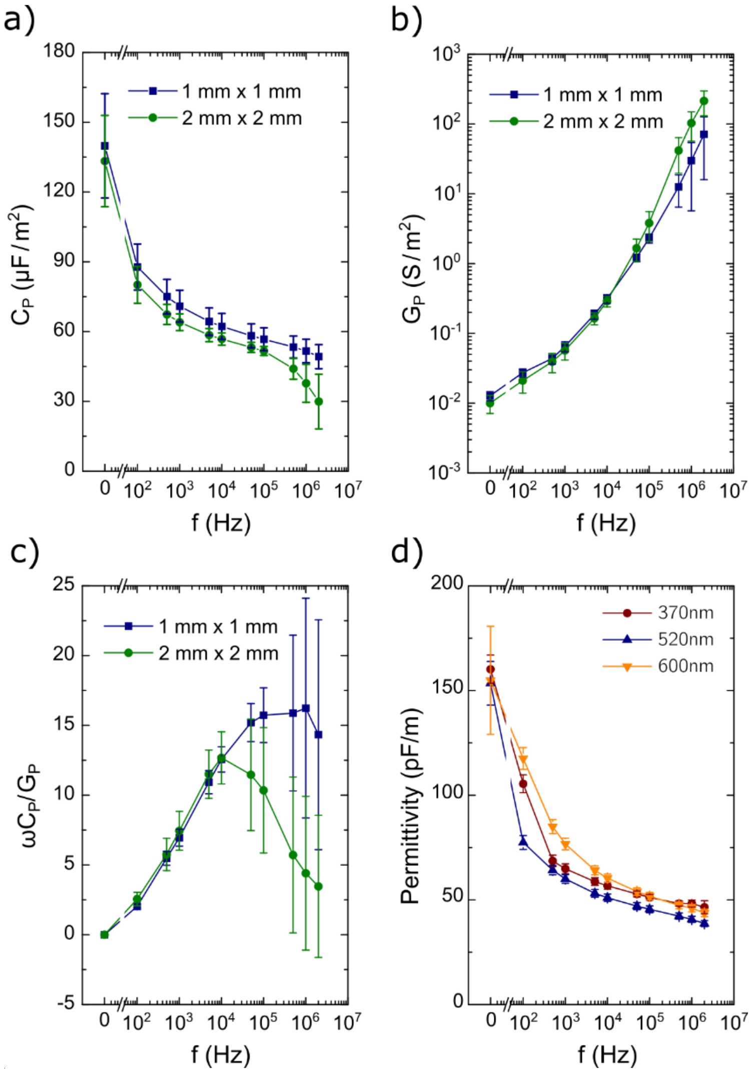

Supplementary Figure 7: Capacitance measurements of albumen thin films. (a) Measured capacitance values per unit area plotted as a function of frequency; (b) measured conductance values per unit area plotted as a function of frequency; (c) reactive over real part ratio of the measured admittance plotted as a function of frequency. All the measured values are referred to 1 mm^2 area (blue line) and 4 mm^2 area (green line) albumen capacitors. The error bars represent the standard deviation of the mean collected values. (d) Permittivity vs. frequency plots for a batch of albumen capacitors with different thicknesses and same top electrode size for thickness dependent capacitance analysis. Thicknesses are labelled in the plot. Error bars for all plots represent standard deviation of the reported values. The graphs report mean values (arithmetic) and standard deviation couples.

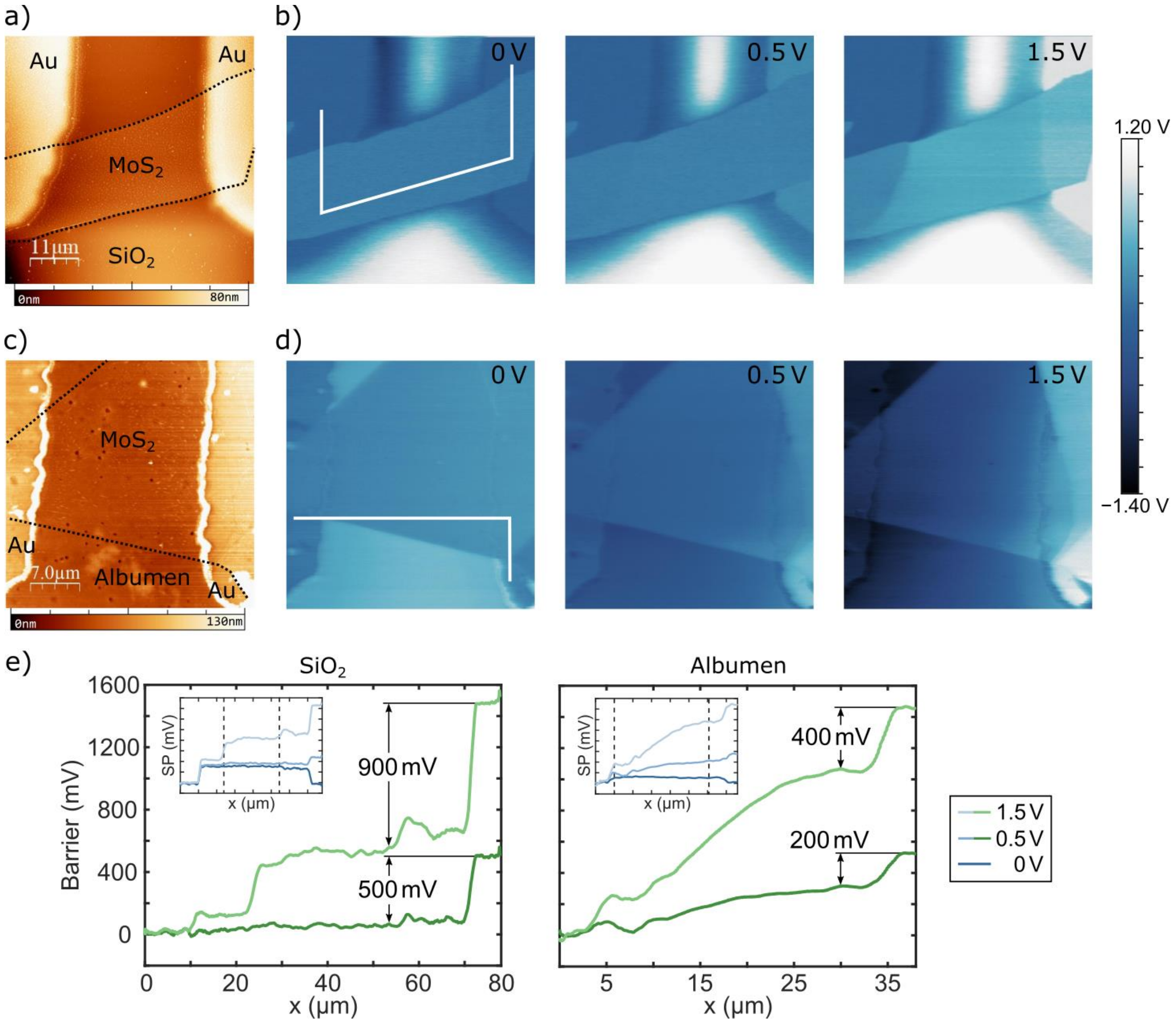

Supplementary Figure 8: KPFM measurements for Schottky barrier analysis. (a) and (c) Recorded topography images of the two devices on SiO_2 and albumen, respectively. (b) and (d) Recorded surface potential maps of both devices for different source-drain biases (0 V, 0.5 V and 1.5 V). The left electrode is grounded. Potential profile lines are indicated in white. (e) Schottky barrier plots for both cases and voltages. The potential profiles extracted from (b) and (d) are shown as insets, with dotted lines marking the MoS_2-area of the profile. The barrier plots result from subtracting the zero-bias profile from the desired voltage profile. The barrier height is the difference between the potential of the gold electrode and the potential of the MoS_2 flake at the border.

Dimensions and electrical device properties of all measured albumen transistors:

Nr.	W (µm)	L (µm)	On/off ratio	g_m (AV^{-1})	Mobility ($cm^2V^{-1}s^{-1}$)	V_{TH} (V)	V_{gm} (V)	n_{2D} (cm^{-2})
6	15	30	3×10^2	6×10^{-7}	56.9	6	7.5	1.97×10^{11}
8	20	25	1.7×10^3	1.3×10^{-7}	7.7	3	6	3.95×10^{11}

9	7	25	5×10^{2}	3.1×10^{-7}	52.5	−3	2	6.58×10^{11}
10	20	25	1×10^{4}	1.5×10^{-6}	88.9	2	5.5	4.61×10^{11}
14	12	35	2×10^{3}	3.5×10^{-7}	48.4	4	6	2.63×10^{11}
16	11	25	3×10^{2}	7.9×10^{-8}	9.1	1	3	2.47×10^{11}
18	7.5	25	2×10^{2}	5×10^{-7}	84.1	6	8.5	3.09×10^{11}
20	20	25	4×10^{3}	1.2×10^{-6}	75.7	6	7	1.24×10^{11}
24*	12	25	7.6×10^{2}	3×10^{-7}	32.0	7	8.5	1.86×10^{11}
25*	11	27	1.5×10^{1}	4.4×10^{-8}	5.5	7	9.5	3.09×10^{11}

Supplementary Table 1: Device geometries and electrical figures of merit of the albumen transistors. Values are extracted for all fabricated albumen transistors. Mobility, transconductance, threshold voltage and transconductance voltage values are corresponding to the forward sweep. Devices 24 and 25 are fabricated with eggwhite extracted from whole eggs, all other devices from bottled pasteurized eggwhite.

Electrical device properties of all measured SiO_2-based transistors:

Nr.	On/off ratio	g_m (AV^{-1})	Mobility ($cm^2V^{-1}s^{-1}$)	V_{TH} (V)	V_{gm} (V)	n_{2D} (cm^{-2})
1	1.35×10^{3}	5×10^{-9}	0.4	−33	19	3.86×10^{12}
2	2×10^{1}	8×10^{-11}	0.002	−50	0	3.71×10^{12}
3	2.5×10^{2}	8×10^{-10}	0.037	−25	21	3.42×10^{12}
4	2.35×10^{3}	1.3×10^{-9}	0.08	−19	18	2.75×10^{12}
5	5.75	5.85×10^{-11}	0.012	−26	29	4.09×10^{12}
6	3.7	1.8×10^{-11}	0.002	−30	6	2.67×10^{12}
7	3.75×10^{3}	5.67×10^{-10}	0.01	−26	1	2.01×10^{12}
8	9×10^{1}	2.69×10^{-10}	0.011	−16	28	3.27×10^{12}
9	5	1.02×10^{-10}	0.003	−14	18	2.38×10^{12}
10	3×10^{1}	7.36×10^{-12}	0.0004	−5	19	1.78×10^{12}
11	4×10^{1}	3.44×10^{-10}	0.021	−10	9	1.41×10^{12}
12	2.7×10^{2}	5.48×10^{-11}	0.065	−19	13	2.38×10^{12}
13	5	2.03×10^{-10}	0.01	−20	16	2.67×10^{12}
14	1.6×10^{4}	4.18×10^{-9}	0.033	−24	−1	1.71×10^{12}
15	1.65×10^{3}	3.87×10^{-10}	0.023	−22	16	2.82×10^{12}
16	1.5×10^{2}	7.36×10^{-12}	0.004	−5	16	1.56×10^{12}

Supplementary Table 2: Electrical figures of merit of the SiO_2-based transistors. Values are extracted for all fabricated SiO_2-based transistors. Mobility, transconductance, threshold voltage and transconductance voltage values are corresponding to the forward sweep.

In the following the characteristic electrical measurements for all albumen devices are given, with the (a) panels showing the current *vs*. voltage (*IV)* curves for gate voltage sweeps of −10

to 10 V and the (b) panels showing the corresponding transfer curves in linear and logarithmic fashion for the same gate bias range. Numbers correspond to the label in Supplementary Table 1. The curves of the albumen transistor with the number 9 are displayed in the main text.

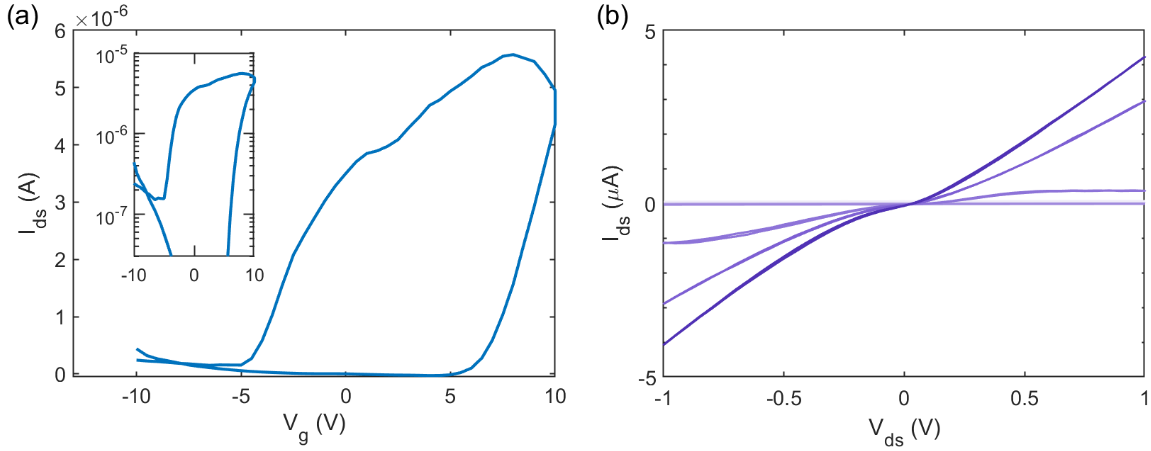

Supplementary Figure 9: Electrical measurements of albumen transistor #6.

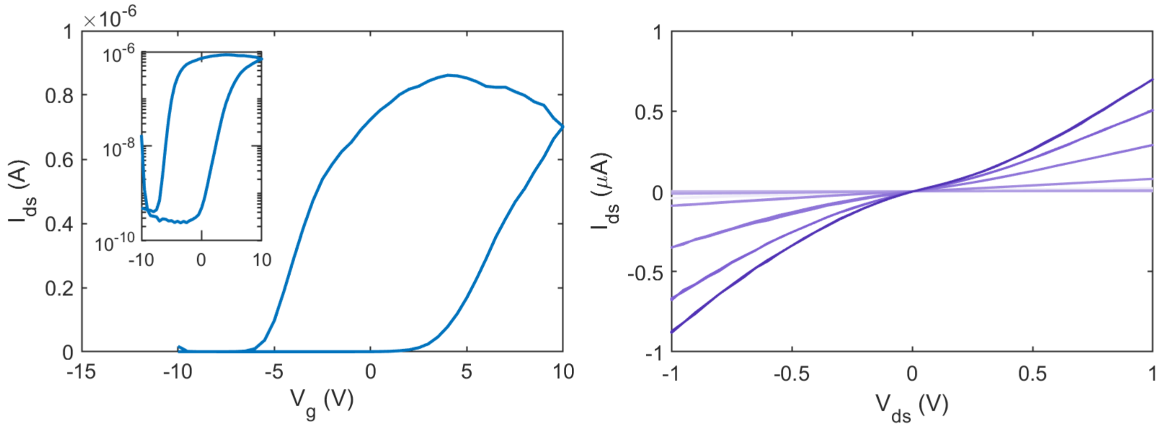

Supplementary Figure 10: Electrical measurements of albumen transistor #8.

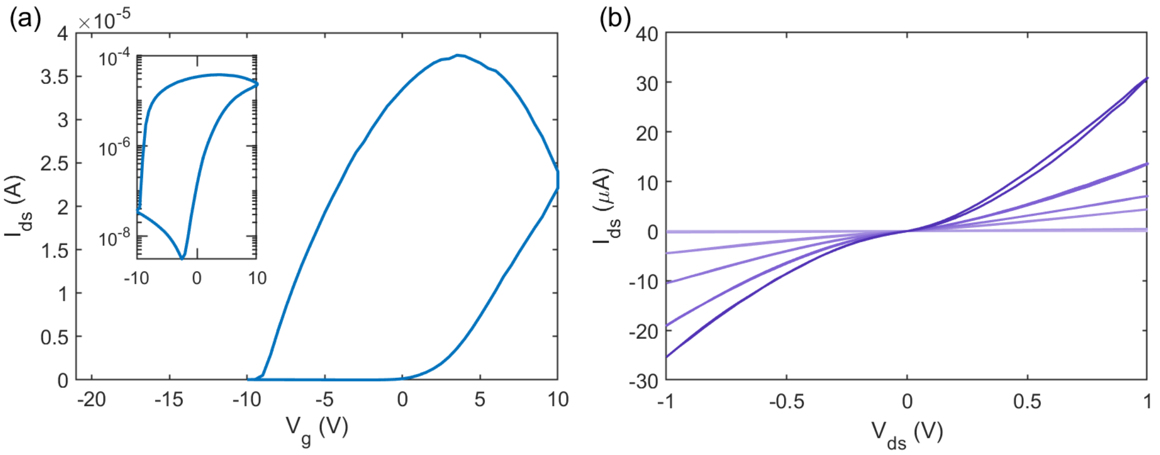

Supplementary Figure 11: Electrical measurements of albumen transistor #10.

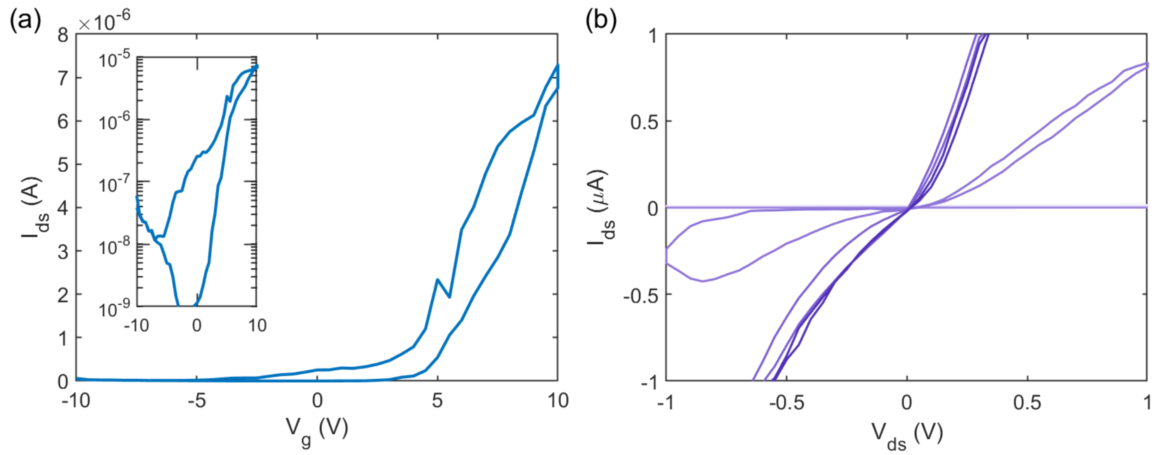

Supplementary Figure 12: Electrical measurements of albumen transistor #14.

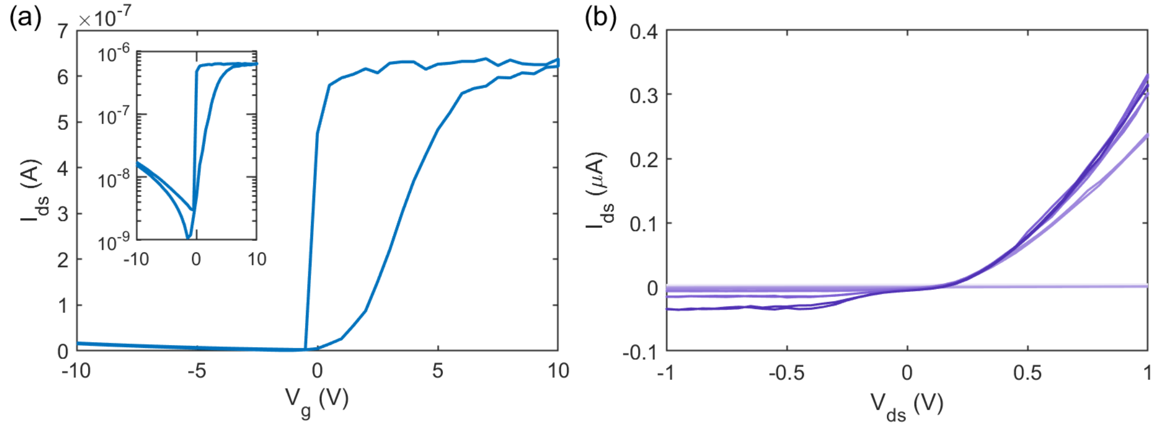

Supplementary Figure 13: Electrical measurements of albumen transistor #16.

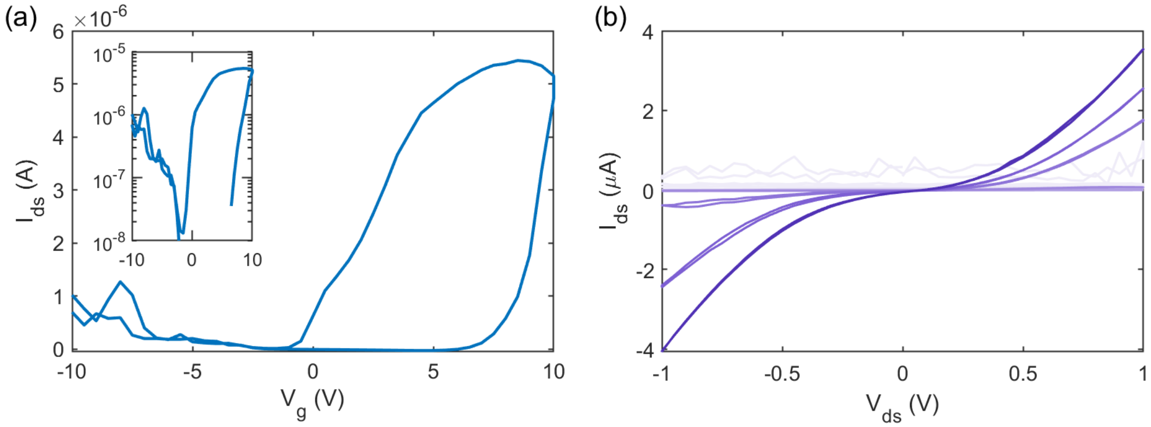

Supplementary Figure 14: Electrical measurements of albumen transistor #18.

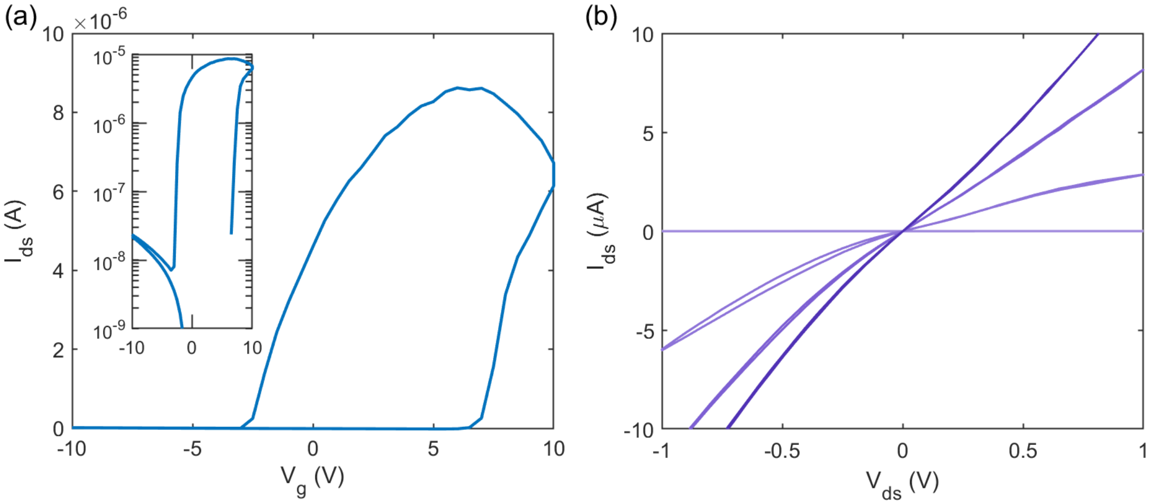

Supplementary Figure 15: Electrical measurements of albumen transistor #20.

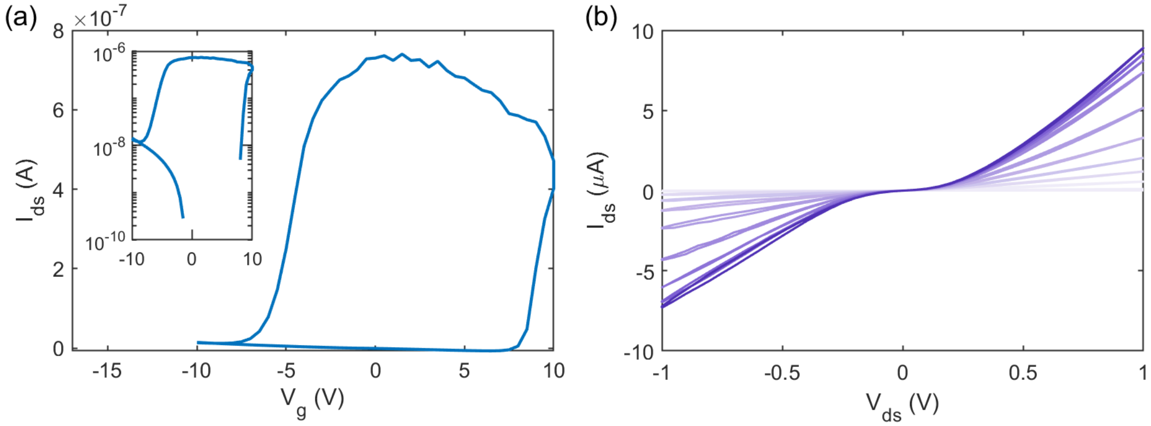

Supplementary Figure 16: Electrical measurements of albumen transistor #24.

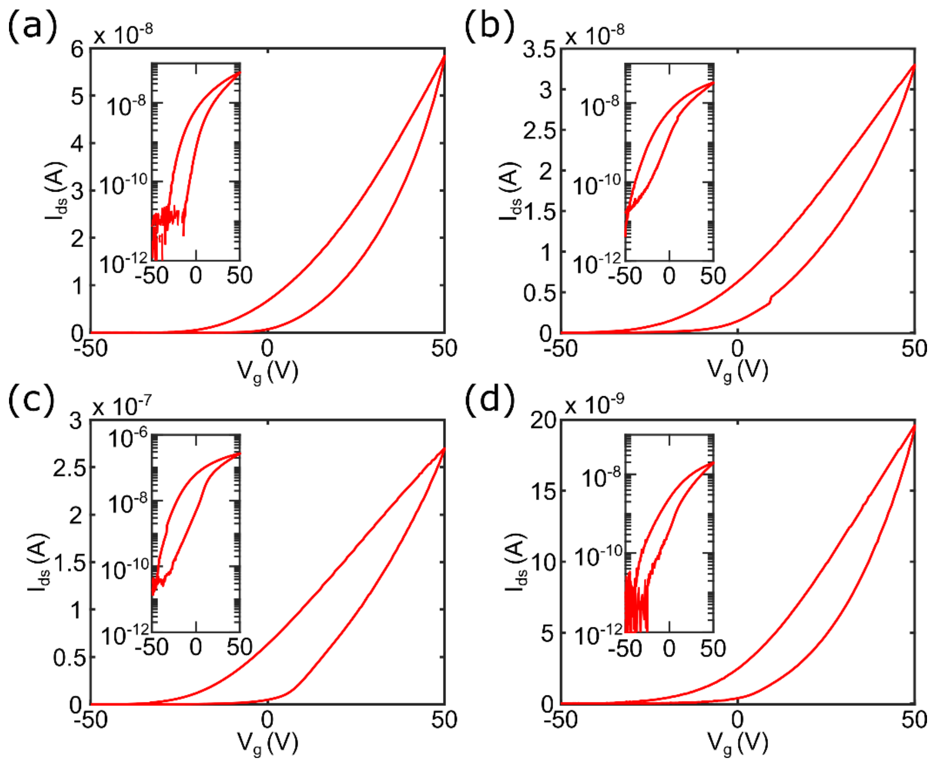

Supplementary Figure 17: Transfer curves of selected SiO_2-based devices. Panels (a)-(d) show the transfer curves of devices 4, 7, 14 and 15, respectively, corresponding to the labels in Supplementary Table 2. The insets show the curves in semi-logarithmic plots. Source-drain voltages for all measurements are V_{ds} = 1V. All curves show clockwise hysteresis.

To confirm the use of only albumen from the whole egg and neglect the yolk, whole egg dielectric transistors were fabricated. Fabrication steps are exactly the same as before, but this time using the liquid parts of both the albumen and the yolk of a whole chicken egg and stirring them until fully combined (Supplementary Figure 18a). The finished transistor is depicted in Supplementary Figure 18b and shows the MoS_2 channel between source-drain electrodes on top of two layers of egg spin-coated subsequently. The egg layer is visually more cracked after fabrication than it was the case for solely albumen. Electrical analysis

shows the possibility of tuning the channel but also reveals big amounts of gate leakage, seen in the current vs. voltage characteristics as an offset voltage (Supplementary Figure 18c). Subtracting the offset from the data it is possible to plot the transfer curve of the egg transistors (Supplementary Figure 18d). Though the FET does not show optimal behavior it is still possible to measure photo response at zero gate voltage. The corresponding measurements are depicted in Fig. S18e and once again indicate MoS_2's excitonic A and B peaks.

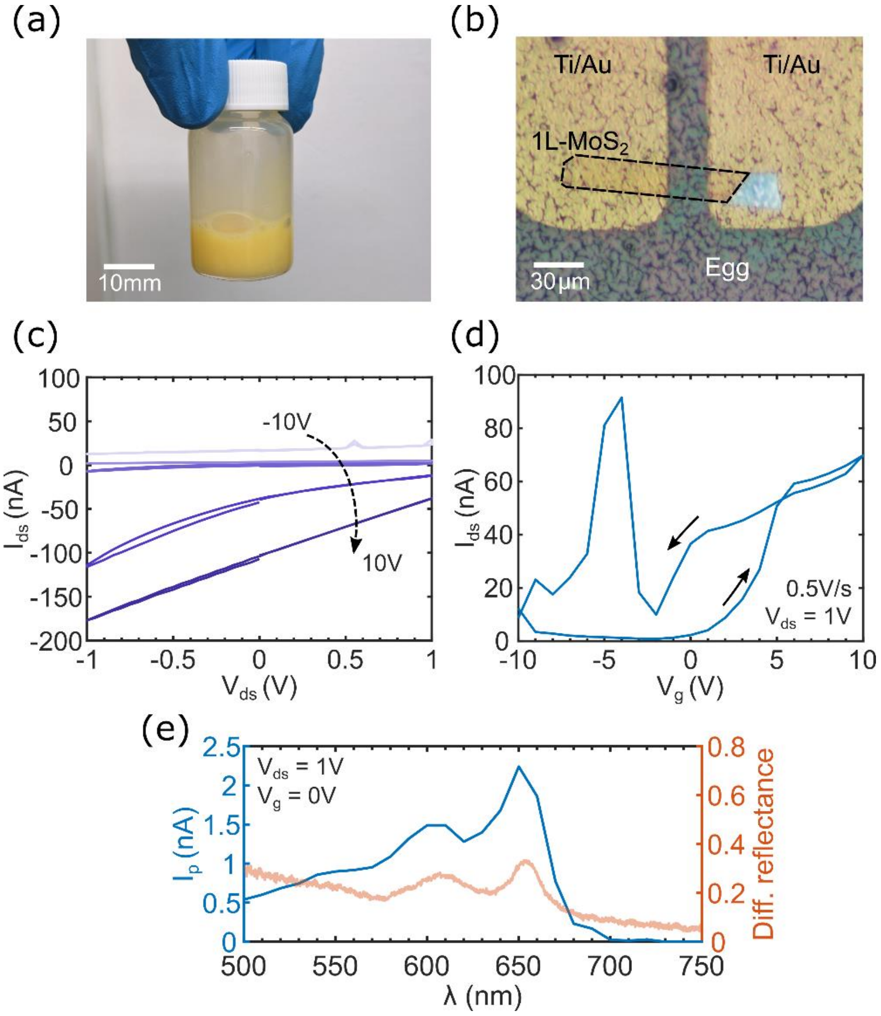

Supplementary Figure 18: Phototransistors using complete egg dielectric. (a) Chosen dielectric material out of whole chicken eggs (mixture of the liquid parts of eggwhite and yolk). (b) Optical micrograph of the finished device after MoS_2 transfer. The baked egg dielectric separates the silicon back gate from the semiconductor channel. (c) Current vs. voltage curves (*IVs*) for different gate

voltages ranging from −10 to 10V. Gate leakage can be observed as an offset voltage from zero. (d) Transfer curves (I_{sd} vs. V_g) plotted in linear fashion. Note that for the transfer curve the leakage offset (seen in (c)) was subtracted to depict the behavior more clearly. (e) Wavelength dependence of the photocurrent showing an enhanced photoresponse at the MoS_2 excitonic energies. For comparison the measured differential reflectance spectrum of the MoS_2 flake prior transfer is added to the plot, relating A (660 nm) and B (610 nm) exciton peaks to the peak responses in photocurrent measurements.
